# Evidence for Cryptic Diversity in the “Pan-Antarctic” Springtail *Friesea antarctica* and the Description of Two New Species

**DOI:** 10.3390/insects11030141

**Published:** 2020-02-25

**Authors:** Antonio Carapelli, Penelope Greenslade, Francesco Nardi, Chiara Leo, Peter Convey, Francesco Frati, Pietro Paolo Fanciulli

**Affiliations:** 1 Department of Life Sciences, University of Siena, Via A. Moro 2, 53100 Siena, Italy; francesco.nardi@unisi.it (F.N.); leo6@student.unisi.it (C.L.); francesco.frati@unisi.it (F.F.); paolo.fanciulli@unisi.it (P.P.F.); 2 Environmental Management, School of Health and Life Sciences, Federation University, Ballarat, VIC 3350, Australia; p.greenslade@federation.edu.au; 3 British Antarctic Survey, NERC, High Cross, Madingley Road, Cambridge CB3 0ET, UK; pcon@bas.ac.uk

**Keywords:** Victoria Land, biogeography, invertebrate biota, chaetotaxy, integrative taxonomy, molecular phylogeny, species delimitation, *Friesea gretae* sp. nov., *Friesea propria* sp. nov.

## Abstract

The invertebrate terrestrial fauna of Antarctica is being investigated with increasing interest to discover how life interacts with the extreme polar environment and how millions of years of evolution have shaped their biodiversity. Classical taxonomic approaches, complemented by molecular tools, are improving our understanding of the systematic relationships of some species, changing the nomenclature of taxa and challenging the taxonomic status of others. The springtail *Friesea grisea* has previously been described as the only species with a “pan-Antarctic” distribution. However, recent genetic comparisons have pointed to another scenario. The latest morphological study has confined *F. grisea* to the sub-Antarctic island of South Georgia, from which it was originally described, and resurrected *F. antarctica* as a congeneric species occurring on the continental mainland. Molecular data demonstrate that populations of this taxon, ostensibly occurring across Maritime and Continental Antarctica, as well as on some offshore islands, are evolutionarily isolated and divergent and cannot be included within a single species. The present study, combining morphological with molecular data, attempts to validate this hypothesis and challenges the taxonomic status of *F. antarctica,* suggesting that two additional new species, described here as *Friesea gretae* sp. nov. and *Friesea propria* sp. nov., are present in Continental Antarctica.

## 1. Introduction

Human-driven environmental change, of which the long-term consequences are only now being appreciated, currently threatens biodiversity, the stability of ecosystems and the services they provide. Knowledge of biological processes that sustain endangered natural environments is relatively incomplete, especially so for remote and isolated ecosystems, such as those of polar regions. Actions to counteract the impact of global environmental change on threatened ecosystems rely on evaluation of their resilience to disturbance [1]. Simulations to predict the impact of changing abiotic factors have already been designed, although their ability to model future trajectories is impeded by geographical heterogeneity and distinctive biological diversity. In Antarctica, poor knowledge of species diversity and of their dispersal ability/resilience are considered limiting factors that prevent the implementation of sustainable conservation plans [2,3,4,5]. The dispersal abilities of species [6,7] and identification of biologically distinct areas are being addressed in order to develop improved protection strategies [5,8,9,10]. As new strategies need to be employed, a combination of traditional and more recently developed approaches (known as integrative taxonomy) is being used to describe the composition of the terrestrial fauna of polar regions and to increase understanding of their resilience.

From a biogeographic point of view, Antarctica is conventionally divided in three large biogeographic regions, the sub-, Maritime and Continental Antarctic [11,12]. More recently, the “Gressitt line” was recognized as a major biogeographical discontinuity separating the Antarctic Peninsula from the reminder of the continent [13], and 16 Antarctic Conservation Biogeographic Regions (ACBRs) were introduced [8,9,14].

The isolation and subsequent progressive cooling of the Antarctic continent after its separation from the other southern landmasses [15,16] initiated the progressive decline in its terrestrial biodiversity. The expansion of continental-scale ice sheets started around 20 Mya to peak at ~14 Mya. Multiple cooling cycles, in the Pliocene and Pleistocene, leaves, as their legacy, the present impoverished terrestrial fauna [12]. This Antarctic terrestrial biota inhabits the <0.4% of the continent that is ice-free, comprising coastal areas, inland nunataks and mountain ranges and the frigid desert dry valleys in Victoria Land.

The most diverse continental Antarctic invertebrates are springtails and mites, many of which are locally endemic. These groups have a long evolutionary history on the continent and must have survived multiple glacial cycles in refugia [15,17,18,19,20,21]. Consistent with the scale of isolation of Antarctic terrestrial habitats and the limited active dispersal capabilities of their invertebrate biota, most population genetic studies so far performed on Antarctic springtail species have shown moderate levels of intraspecific genetic divergence. For example, [4] reviewed patterns of population structure observed in seven species endemic to Victoria Land and [22] proposed a role for past geological events, at least in Victoria Land. From their data, it was evident that, even though different lineages could be detected with distributions shaped by geographic barriers (e.g., the Tucker, Mawson and MacKay Glaciers), these did not show signs of strong genetic differentiation, at least in four of the seven species (*Kaylathalia klovstadi*, *Cryptopygus cisantarcticus*, *C. nivicolus* and *Gomphiocephalus hodgsoni*). In particular, although *K. klovstadi*, *C. cisantarcticus* and *Friesea antarctica* (referred to as *Friesea grisea*) populations are distributed both north and south of the Tucker glacier, only *F. antarctica* showed particularly high values of pairwise genetic distances (9.2%), whereas the former two species were characterized by genetic divergence (ranging between 1.7% and 5.8% [4]) which are within the range generally observed at the intraspecific level in springtails [23].

In the Antarctic Peninsula, the highest values of genetic diversity were observed between *Cryptopygus antarcticus* populations [7,24], despite high gene flow and limited signs of genetic structuring (after the automatic barcode gap discovery [25]); high values of genetic diversity have also been documented in *Antarctinella monooculata*, *F. antarctica* [26] (as *F. grisea*) and *Cryptopygus terranovus* [27]. In contrast, very low values of genetic divergence were observed in *Folsomotoma octooculata* [2].

The genus *Friesea* dalla Torre 1895 (Collembola: Poduromorpha: Neanuridae: Pseudachorutinae) is the most species-rich and widespread genus of springtails in Antarctica; it currently includes 190 described species globally [28] and has a worldwide distribution. The genus is characterized by the unique form of the maxilla, absence of a post antennal organ, abdomen VI carrying spines or spine-like chaetae and reduced furca. For Antarctic *Friesea grisea* (Schäffer, 1891), there is a plethora of ecological, physiological and taxonomic literature [29,30,31]. This species, whose type locality is on sub-Antarctic South Georgia, has recently been re-described and shown to have a distribution restricted to that island [3] and to be absent from the Antarctic Continent, contrary to earlier opinion. *Friesea* populations on the Antarctic Peninsula and the Antarctic Continent, previously recorded as *F. grisea*, have therefore been designated to the resurrected *F. antarctica* [3], with records from North and South Victoria Land in Eastern Antarctica and various locations along the Antarctic Peninsula, its offshore islands and the South Shetland Islands. Clear morphological distinction is apparent; when compared with *F. antarctica*, *F. grisea* specimens have a paler color and a larger body size, as well as possessing dorsal cuticular plaques (absent in *F. antarctica*) and a different number and shape of both clavate tenant hairs and anal spines [3]. Recently, specimens from near the Russian Molodyozhnaya Station, recorded by Wise [32] as *F. grisea* and Bulavintsev [33] as *Friesea* sp., were found to be another new species, *F. eureka* [34].

The anomalous genetic divergence observed between populations of *F. antarctica* from the Antarctic Peninsula (AP) and Victoria Land (VL) sites (e.g., an overall genetic distance of 21.7% between the mitochondrial genomes from specimens of the two regions [35]) contrasts with an identical morphology [26,35]. The presence of cryptic species hidden within the *F. antarctica* complex was hypothesized and an in-depth morphological revision of the taxon suggested [35]. No molecular data are so far available from the type locality of *F. grisea* to allow comparison. Within *F. antarctica*, population genetic and phylogenetic studies have identified well-defined evolutionary lineages, here referred to as AP (Antarctic Peninsula), VL (Victoria Land) and Cape Hallett, clearly separated from each other in geographical terms. However, as yet, no morphological analyses are available to support a distinction between these three lineages. This may be due to either phylogenetic niche conservatism (e.g., [36]; see Discussion section) or morphological diversity that has been previously overlooked. The current study evaluates their morphology and genetic differentiation through a multidisciplinary comparative analysis. We tested the efficacy of an integrative taxonomic approach to assess whether or not cryptic speciation took place, while continuing morphological uniformity has mainly been selected for by the extreme environmental conditions (i.e., phylogenetic niche conservatism). Morphological revision of *F. antarctica* specimens from both the Antarctic Peninsula and Victoria Land, with specimens from Cape Hallett further compared against both regions, was performed. Morphological data were re-evaluated in the light of molecular data. Different bioinformatic tools for species delimitation, based both on genetic distances and phylogenetic reconstruction, were used to analyze nuclear and mitochondrial markers. Descriptions of two new species, previously included within *F. antarctica* and emerging from both morphological and molecular analyses, are provided.

## 2. Materials and Methods

### 2.1. Sample Collection

**Figure 1:**
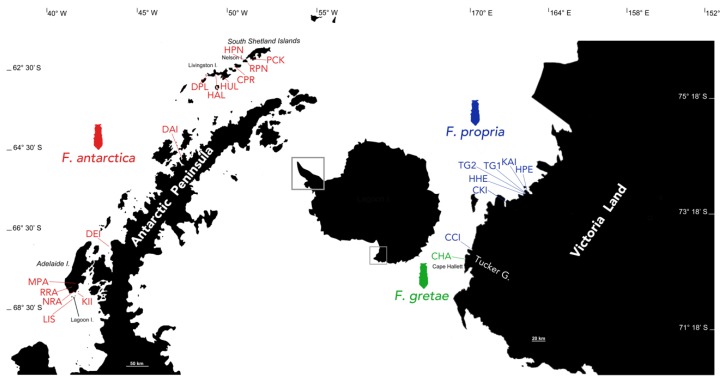
Map of sampling localities for the *Friesea* populations. Different colors identify each proposed species occurring in maritime (red) and continental (blue and green) Antarctica. See Table 1 for collecting site acronyms and geographical locations.

Specimens initially identified as *F. antarctica* (known at that time as *F. grisea*) were collected from both the Antarctic Peninsula and Victoria Land (sampling sites and respective abbreviations given in Figure 1 and Table 1). Specimens from Victoria Land were collected during the Italian National Antarctic Program (PNRA) expeditions (2005/2006, 2017/2018 and 2018/2019). Individuals of *F. antarctica* from the Antarctic Peninsula were sampled during the 2002/2003 expedition through a collaboration between the British Antarctic Survey (BAS) and the PNRA. After their collection from field habitats using a suction device, specimens were subjected to preliminary morphological identification and stored at −80 °C until further morphological and molecular analyses.

### 2.2. Morphological Analyses

Between five and 20 specimens of *F. antarctica*, from the localities highlighted by asterisks in Table 1, were incubated in lactic acid (at 35–45 °C) and cleared by short immersion (2–10 min) in 10% KOH. Samples were mounted on slides and observed under a Leitz Laborlux S microscope. Some other specimens were mounted on slides after clearing in Nesbitt to improve the observation of chetae and spines on Ab. V–VI, as needed. When possible, body length, the cephalic diagonal and antennae, as well as the length and width of each thoracic and abdominal segment, were measured. Since data distributions were not normal, the nonparametric Mann-Whitney test with Bonferroni correction for multiple comparisons was applied to assess whether or not there were significant differences in these parameters, as well as in the number of setae in the male and female genital openings. Calculations were performed using R 3.3.2 [37], comparing AP vs. VL, AP vs. Cape Hallett and VL vs. Cape Hallett specimens (see Results).

Some samples were also prepared for scanning electron microscopy. Firstly, they were dehydrated in absolute ethanol followed by critical-point drying in a Balzer CPD 030. Samples were coated with gold in a Balzer MED 010. Scanning electron microscopy observations were performed using a Philips XL20.

### 2.3. Molecular Dataset

The datasets assembled by Torricelli et al. [26] and Carapelli et al. [2] were enlarged, adding specimens of *F. antarctica* from Danco Island and Detaille Island (Table 1), as well as four outgroup species: the congeneric *F. topo* (from Alexander I.) and *F. salmoni*, as well as *Bilobella aurantiaca* and *Folsomotoma octooculata*. All specimens were screened not only for two previously obtained mitochondrial markers (*cox1* and *atp6* [2,26]) but also for two further nuclear markers (*28S* and *EF-1α*; large ribosomal subunit and elongation factor subunit 1α, respectively).

Whole genomic DNA was extracted from *F. antarctica* and outgroup specimens using the Wizard^®^ SV genomic DNA Purification System (Promega, Madison, WI, USA) and eluted in 50 μl ddH_2_O. The nuclear markers analyzed in the present study (*28S* and *EF-1α*) were amplified and sequenced with universal primer pairs. The oligonucleotides D1a [5′ CCCSCGTAAYTTAAGCATAT 3′] and D7b [5′ GACTTCCCTTACCTACAT 3′], as well as the nested primers D3a [5′ GACCCGTCTTGAAACACGGA 3′] and D5b1 [5′ ACACACTCCTTAGCGGA 3′] were used to amplify and sequence the large ribosomal subunit (28S rDNA). Specific primers (sequence available on request), matching the flanking portions of the deleted regions of the *28S* ribosomal RNA (*rRNA*) pseudogene, were tested for their ability to amplify this DNA fragment in all samples. The primers M3 [5′ CACATYAACATTGTCGTSATYGG 3′] and rcM4 [5′ ACAGCVACKGTYTGYCTCATRTC 3′], as well as the nested M44.1 [5′ GCTGAGCGYGARCGTGGTATCAC 3′] and rcM51.1 [5′ CATRTTGTCKCCGTGCCAKCC 3′], were applied to amplify and sequence the *EF-1α*. PCRs were performed in a 25 μL reaction volume consisting of: 2.5 μL of whole genomic DNA from each sample, 1.25 μL of both forward and reverse primers (10 μM), 12.5 μL of GoTaq^®^ Long PCR Master Mix (Promega, Madison, WI, USA) and 7.5 μL of ddH_2_O. Amplifications were run on a GeneAmp^®^ PCR System 2700 (Applied Biosystems, Foster City, CA, USA) thermal cycler. The initial denaturation step was set at 95 °C for 5 min, followed by 35 cycles at: 94 °C for 1 min, 50–55 °C (depending on marker) for 1 min and 60 °C for 90 s. The final elongation step was set at 72 °C for 7 min. PCR products were then purified with the kit Wizard^®^ SV Gel and PCR Clean-up (Promega, Madison, WI, USA) and sequenced on both strands with a DNA Analyzer ABI 3730 at the core facility of the Biofab Research Lab (Rome, Italy). The sequences were then manually corrected and assembled using the software Sequencher 4.4.2 (Gene Codes Corporation, Ann Arbor, MI, USA). For each of the nuclear and mitochondrial markers, the dataset was aligned using the online tool Clustal Omega (https://www.ebi.ac.uk/Tools/msa/clustalo/) and manually corrected in Mesquite [38]. This latter program was further applied to concatenate the four loci into a multi-locus dataset. The four single- and the multi-locus alignments were then used for phylogenetic and species delimitation analyses.

### 2.4. Phylogenetic Analyses

Maximum likelihood (ML) and Bayesian inference (BI) analyses were performed on the four single- and the multi-locus datasets, as implemented in IQ-TREE v1.6.0 [39] and in MrBayes v3.2 [40], respectively.

For both phylogenetic optimization criteria, the best models of evolution were selected before the tree search, considering different dataset partitions: 1st; 2nd and 3rd codon positions for the protein-encoding genes (*cox1*, *atp6* and *EF-1α*) and one single partition was considered for the *28S*. The *EF-1α* dataset was further partitioned into coding and noncoding regions (i.e., the three codon positions, as well as the introns) (models of evolution applied in the present study are summarized in Table 2).

For ML analyses, the best models of evolution were detected through ModelFinder [41] based on the Bayesian information criterion (BIC) and a greedy strategy. The ML tree searches were performed with various settings of perturbation strength (-pers command) of 0.2; 0.5; 0.7 and stop condition (-nstop command) of 100, 200 and 300. The support values were obtained with 1000 replicates of ultrafast bootstrap approximation [42].

Before the BI analyses, the best models of evolution were selected by means of the software PartitionFinder v2.1 [43] based on the Akaike’s information criterion (AIC) and a greedy strategy. The models of evolution that best fitted our datasets were then applied to MrBayes 3.2, run with four chains (three hot and one cold) for 10^6^ generations with a sampling frequency of one tree every 1000 iterations and the first 25% of the tree topologies discarded (burn-in step).

### 2.5. Species Delimitation Analyses

Given the high genetic differentiation, previously detected through mitogenomic and population genetic studies [26,35], and the morphological uniformity of the *F. antarctica* specimens, several methods of species delimitation based on molecular markers were applied. Both the single- (*cox1*, *atp6*, *28S* and *EF-1α* genes, individually aligned) and multi-locus (concatenation of *cox1*, *atp6*, *EF-1α* and *28S* loci) datasets were tested, starting with species discovery approaches (see Section 2.5.1, Section 2.5.2 and Section 2.5.3 below). Then, putative species clusters detected were further investigated through a validation method of species delimitation (see Section 2.5.4 below). Both distance- and phylogeny-based approaches were used.

#### 2.5.1. Distance-Based Species Delimitation

The *cox1* single-locus dataset was processed with the online tool automatic barcode gap discovery (http://wwwabi.snv.jussieu.fr/public/abgd/abgdweb.html [25]). The analysis was performed with default settings (relative gap width of 1.5; intraspecific divergence: between 0.001 and 0.1), as well as applying the Kimura two-parameter (K2P) model when calculating the distribution of pairwise distances.

#### 2.5.2. Phylogeny-based methods: the Poisson Tree Process

The identification of species boundaries was also carried out using phylogenetic methods, based either on the number of substitutions (bPTP [44]) or on divergence time estimations (GMYC [45]; see Section 2.5.3). Since both branch length (that may reflect the number of nucleotide substitutions) and the topology depend on the optimization criteria applied to the inference analysis, the four single-locus datasets were used for both BI and ML tree search, as described above. The two topologies obtained for each of the four molecular markers were then analyzed through the Poisson Tree Process (PTP) on the web server bPTP (https://species.h-its.org/ptp/ [44]). The analyses were run for 500,000 Markov chain Monte Carlo (MCMC) generations and with a burn-in value of 0.25, both including and excluding the outgroup species.

#### 2.5.3. Phylogeny-Based Methods: The Generalized Mixed Yule Coalescent Model

In order to define species boundaries according to the divergence time estimations, the *cox1* dataset was run with the software BEAST 2.4.8 [46], thus obtaining an ultrametric tree. The software PartitionFinder v2.1 was applied to detect the models of evolution that better fitted our single-locus dataset, organized into only one charset. A strict molecular clock was applied to the *cox1* alignment, defining the clock rate based on the average mutation rate per million years identified in Brower [47] and Papadopoulou [48], with a coalescent model of constant population size as tree prior. Two independent MCMC runs were performed, constituting of 10^6^ generations with parameters sampled every 1000 iterations and a burn-in of 0.25. The two runs were combined through the BEAST package LogCombiner 2.4.8 [46]. The convergence of MCMC chains was assessed with Tracer v 1.7 [49] and the tree visualized through the software FigTree v1.4.3 (http://tree.bio.ed.ac.uk/software/figtree/).

The ultrametric topology, obtained as outlined before, was then used as an input for the single-threshold GMYC analysis [45]. This latter was conducted in R 3.3.2 [37] with the use of the splits package [50].

#### 2.5.4. Validation Approach of Species Delimitation

The hypothetical species boundaries detected were then tested and validated under the multispecies coalescent model, by means of the software BP & P (Bayesian Phylogenetic & Phylogeographic [51]). Both the single- (*cox1*, *atp6*, *28S* and *EF-1α* genes individually aligned) and the multi-locus datasets were used for the validation analyses of species delimitation. For each analysis, joint species delimitation and species-tree inference were performed (i.e., speciesdelimitation = 1 and speciestree = 1; A11 [51]). The algorithm 0 and the default settings for fine-tuning parameters were used (ε = 5), as well as the species model prior 1 (uniform probability for rooted tree). The species delimitation analyses were run with the following combination of gamma distributions of the parameters θ (ancestral population size) and τ (root age): (1) θ:G (1:10), τ:G (1:10); (2) θ:G (2:100), τ:G (2:500) and (3) θ:G (2:1000), τ:G (2:200). The analyses were run for 100,000 MCMC generations, with a sampling frequency of 50 and a burn-in of 1000 generations; each analysis was run twice in order to confirm the consistency of the results.

## 3. Results

### 3.1. Morphological Study

#### 3.1.1. Systematics 

Characters that have been considered species-specific in the past for the genus are: number of ocelli, number of clavate tenent hairs, degree of reduction and form of the furca, number of teeth on the retinaculum, number and position of s chaetae on antennal segment IV and number of anal spines [3,52]. 

**Figure 2:**
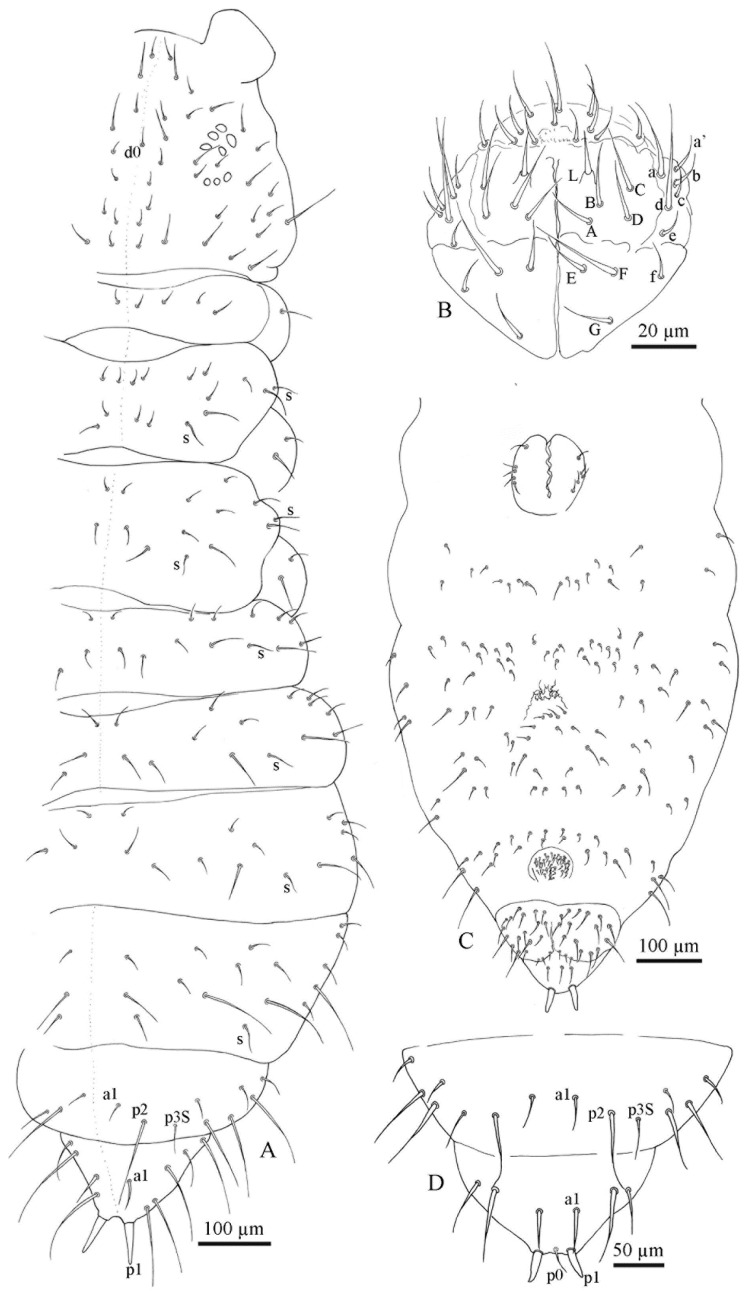
*Friesea antarctica*. (**A**) Dorsal chaetotaxy, (**B**) chaetotaxy of labrum and labium, (**C**) ventral chaetotaxy and (**D**) dorsal chaetotaxy of Abd. V–VI.

**Figure 3:**
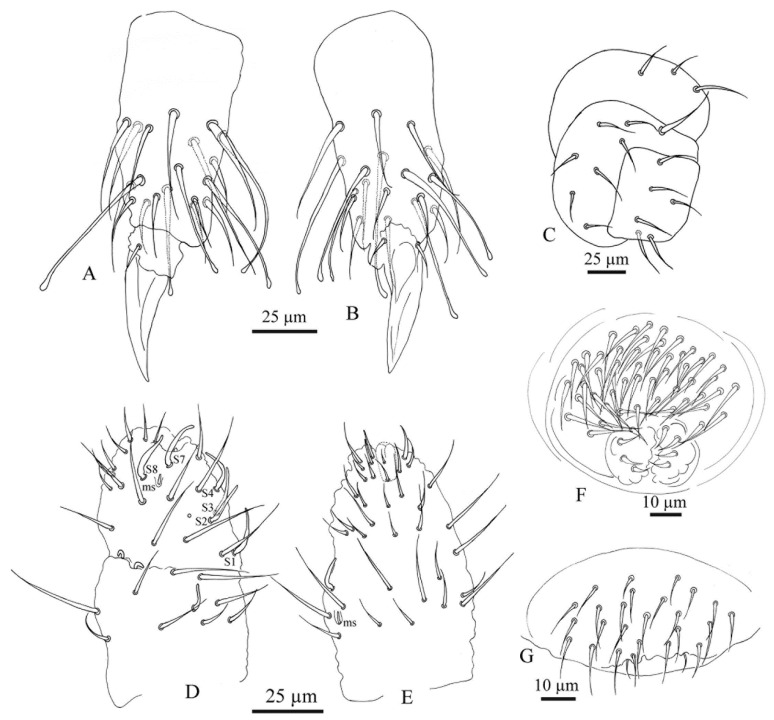
*Friesea antarctica*. (**A**) Chaetotaxy of tibiotarsus I; (**B**) chaetotaxy of tibiotarsus III; (**C**) chaetotaxy of precoxae 2, coxa and trochanter; (**D**) dorsal chaetotaxy of Ant. III–IV; (**E**) ventral chaetotaxy of Ant. III–IV; (**F**) male genital opening and (**G**) female genital opening.

**Figure 4:**
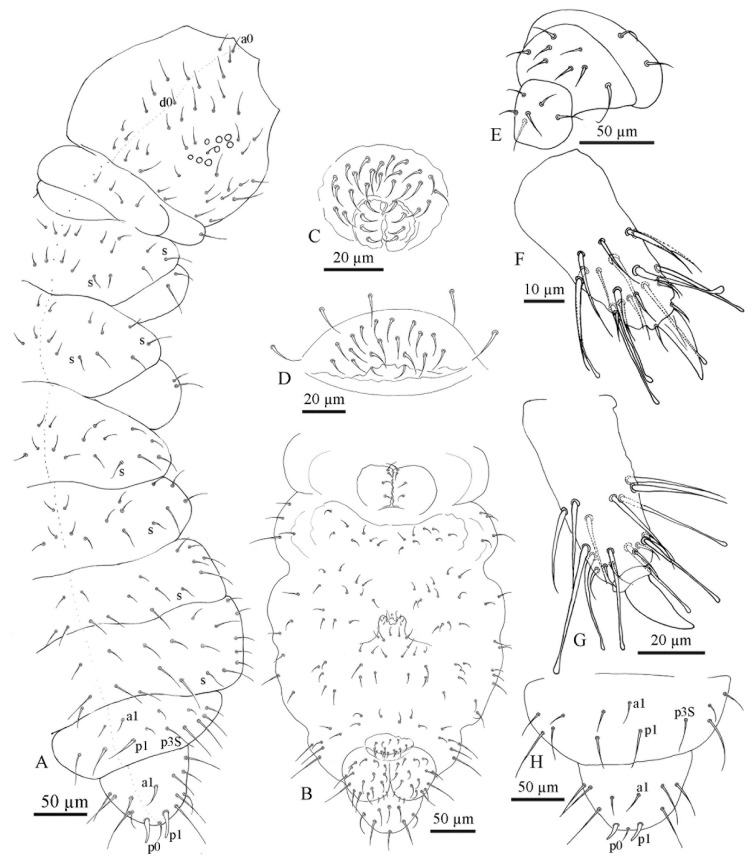
*Friesea propria* sp. nov. (**A**) Dorsal chaetotaxy, (**B**) ventral chaetotaxy; (**C**) male genital opening; (**D**) female genital opening; (**E**) chaetotaxy of precoxae 2, coxa and trochanter; (**F**) chaetotaxy of tibiotarsus I; (**G**) chaetotaxy of tibiotarsus III and (**H**) dorsal chaetotaxy of Abd. V–VI.

**Figure 5:**
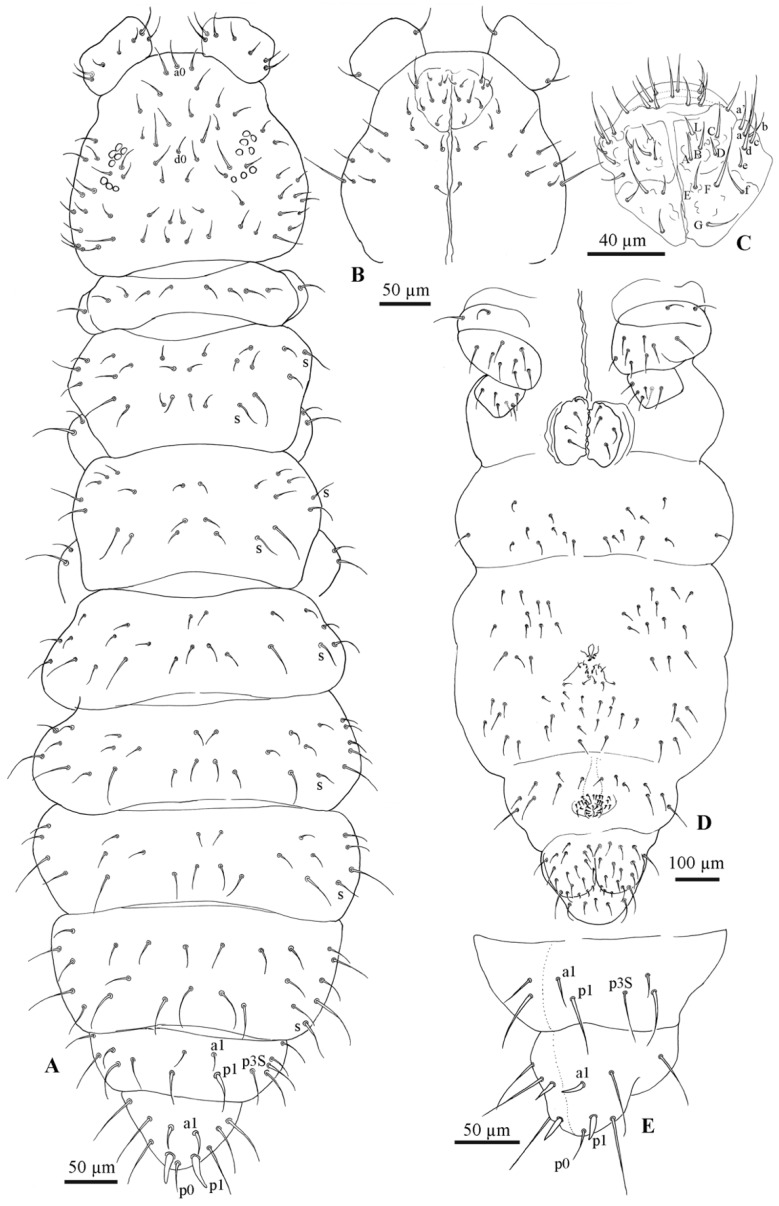
*Friesea gretae* sp. nov. (**A**) Dorsal chaetotaxy, (**B**) ventral chaetotaxy of the head, (**C**) chaetotaxy of labrum and labium, (**D**) ventral chaetotaxy of the body and (**E**) dorsal chaetotaxy of Abd. V–VI.

**Figure 6:**
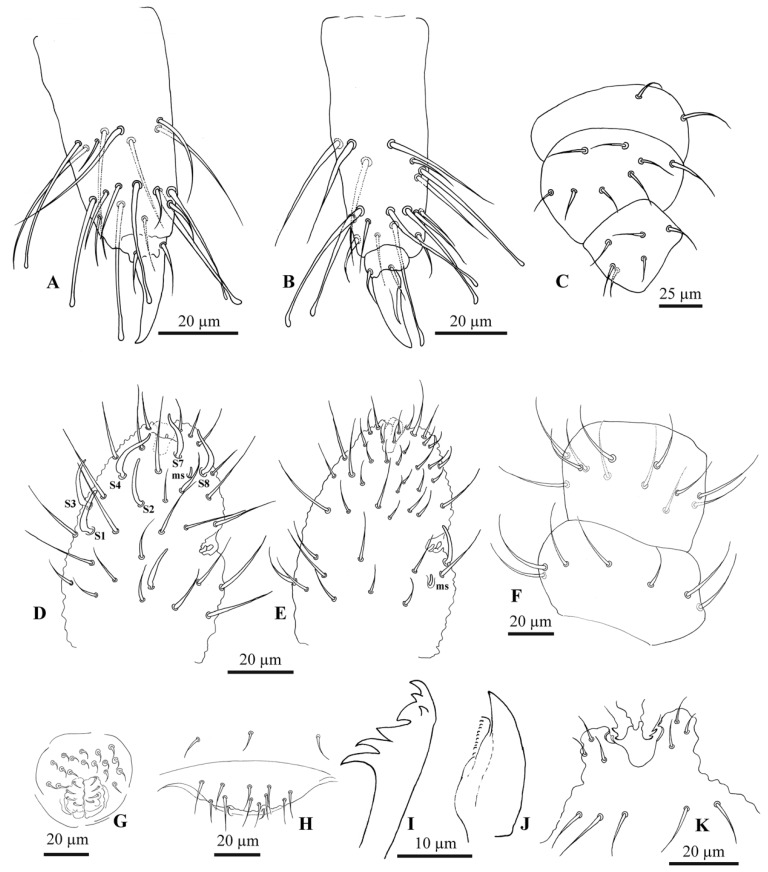
*Friesea gretae* sp. nov. (**A**) Chaetotaxy of tibiotarsus I; (**B**) chaetotaxy of tibiotarsus III; (**C**) chaetotaxy of precoxae 2, coxa and trochanter; (**D**) dorsal chaetotaxy of Ant. III–IV; (**E**) ventral chaetotaxy of Ant. III–IV; (**F**) chaetotaxy of Ant. I–II; (**G**) male genital opening; (**H**) female genital opening; (**I**) mandible; (**J**) maxillae and (**K**) furca and tenacle.

**Figure 7:**
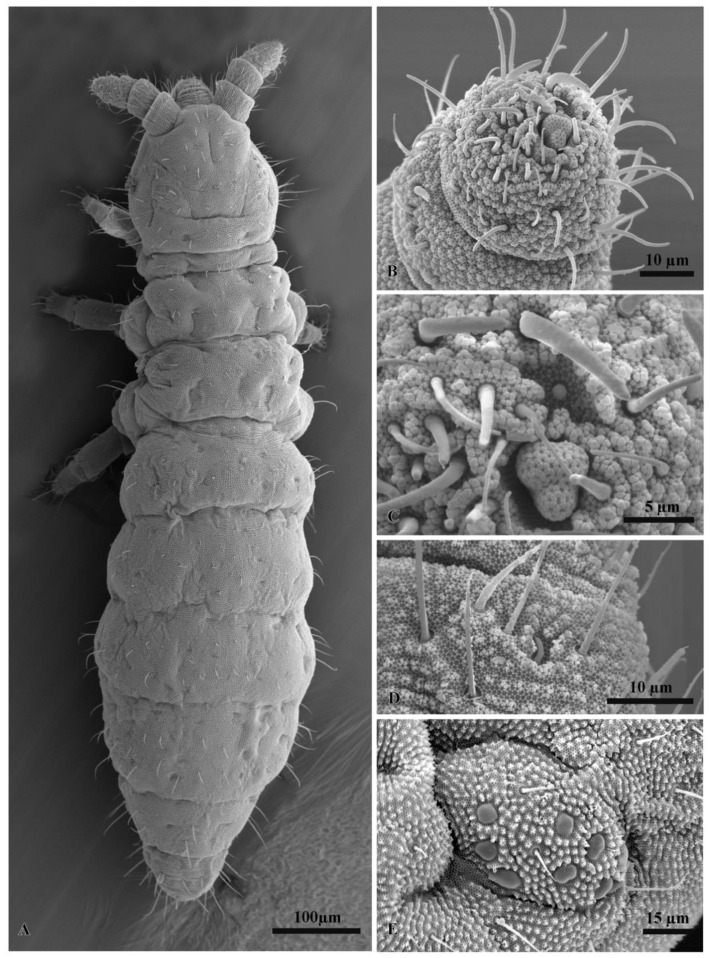
Scanning electron microscopy. (**A**) Dorsal view of *Friesea propria* from Crater Cirque, (**B**) chaetotaxy of Ant. IV in *F. antarctica* from Reptile Ridge (Adelaide I), (**C**) apical vesicle and organite of Ant. IV in *Friesea antarctica* from Reptile Ridge (Adelaide I), (**D**) ventral microsensillum of Ant. III in *Friesea gretae* from Cape Hallett and (**E**) eye spot with 8 ocelli in *Friesea propria* from Crater Cirque.

**Figure 8:**
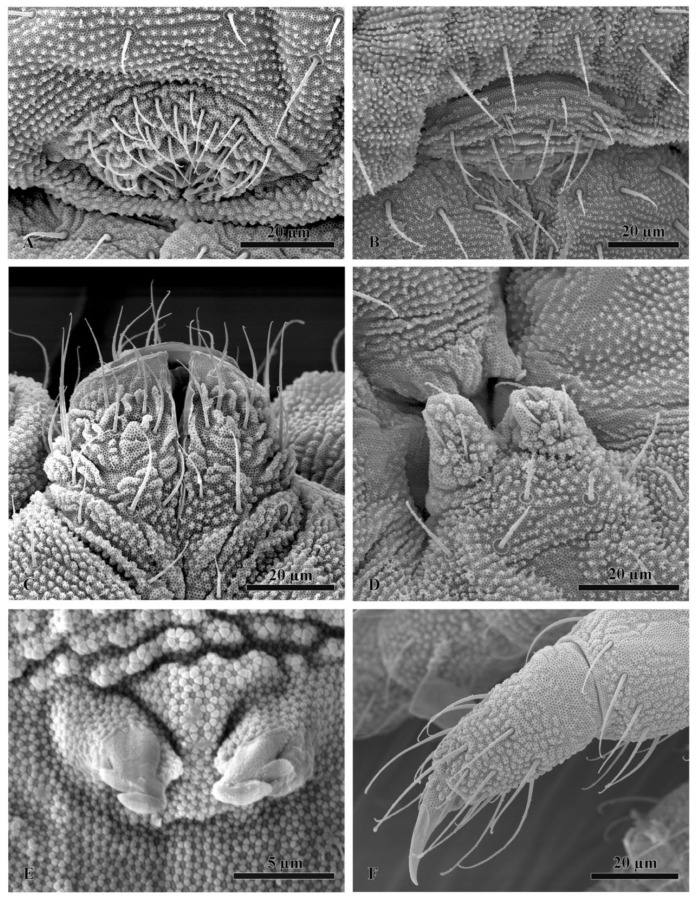
Scanning electron microscopy. (**A**) Male genital opening of *Friesea propria* from Crater Cirque, (**B**) female genital opening in *Friesea gretae* from Cape Hallett, (**C**) chaetotaxy of labium in *Friesea propria* from Crater Cirque, (**D**) furca and tenaculum in *Friesea gretae*, (**E**) tenaculum of *Friesea antarctica* from Reptile Ridge (Adelaide I.) and (**F**) tibiotarsus II of *Friesea antarctica*, specimen from Reptile Ridge (Adelaide I.).

All specimens of the *F. grisea* complex are identical, as far as number of ocelli and form of furca are concerned. We have found the following additional characters useful in distinguishing species: the relative lengths and form of the chaetae on the body, particularly on abdomen V and VI; whether spines, spine-like or normal chaetae are present on abdomen VI; the relative lengths of s chaetae and ordinary and macrochaetae on abdomen V, as well as the presence of dorsal cuticular granulation, body size and colour of adults and development of clavate tenent hairs and clavate macrochaetae. In spite of some intra–population variability, the reliability of these characters is reinforced by the number of specimens we have examined and by the observed consistency with the results of molecular analyses. Line drawings are presented for each species (*F. antarctica*: Figure 2 and Figure 3; *F. propria*: Figure 4; *F. gretae*: Figure 5 and Figure 6), scanning electron microscope details are provided below (Figure 7 and Figure 8).

#### 3.1.2. Morphological Description

Friesea antarctica (Willem, 1901).

= *Achorutoides antarcticus* (Willem, 1901 and 1902).

= *Pseudotullbergia grisea* (Willem, 1901) partem: syn. Wahlgren 1906.

Type Locality: Holotype from Harry Island, Gerlache Straits (presumed lost)

Willem [53,54] described *A. antarcticus* using materials from Harry Island, Gerlache Straits, erecting a new genus for it, *Achorutoides*. It was later synonymized by [55] with *F. grisea.* It was re-erected by Greenslade [3] for specimens from the Antarctic Peninsula and South Shetland Islands. Willem’s description and figures [54] are detailed and accurate except for minute details of the mouthparts. We add descriptions of the chaetotaxy and give sizes and ratios of chaetal lengths here.

The original type material cannot be now found. It is probable that Willem’s original collection was deposited at Ghent University, Belgium, although the author did not indicate where his material was deposited, as it was not customary at that time. Nevertheless, efforts to trace it failed, and the type material probably no longer exists. Thus, according to Article 75.3.4 of the International Code of Zoological Nomenclature, a neotype is erected here from a location as near as possible to the original type locality, Brabant Island. The original specimen was collected on Gerlache’s Belgian expedition in the ship Belgica, who explored the Gerlache Straits and its islands in January 1898.

Neotype: Male, Brabant Island, Gerlache Straits, NN26, MB. BAS, 3.iii.1984. South Australia Museum.

Paraneotype: Female, same collection data. South Australia Museum.

##### Material Examined for Re-description

Potter Cove, King George Island 3 slides (3 ♂); Devils Point, Livingston Island 6 slides (4 ♀, 1 ♂); Hurd Peninsula, Livingston Island 3 slides (♂); Hannah Point, Livingston Island 2 slides (1 ♀, 1 ♂); Livingston Island 20 slides (6 ♀, 7 ♂, 7 ind.); Coppermine Peninsula, Robert Island 3 slides (3 ♂); Harmony Point, Nelson Island 4 slides (3 ♀, 1 ♂); Deception Island 5 slides (1 ♀, 4 ♂); Primavera Cape, San Martín Land 3 slides (1 ♀, 2 ♂); North Point, Rothera, Adelaide Island 4 slides (1 ♀, 1 ♂, 2 ind.); Reptile Ridge, Adelaide Island 5 slides (2 ♀, 3 ♂); Margarite Bay, Léonie Island, 9 slides (6 ♀, 3 ♂) and Lagoon Island 12 slides (1 ♀, 4 ♂, 7 ind.). See Table 1 for geographical details of collecting sites. Specimens will be conserved in the South Australia Museum and in the Collembola Collection of the Department of Life Sciences in the University of Siena.

Habitus size and colour: Neotype 1.8 mm and Paraneotype 1.9 mm, both types on same slide. 

Average length 1.55 mm, same in males and females, range from 1.16 mm to 2.00 mm. Original body length in Willem = 1.4 mm [54].

Body color dark blue, grey, sometimes lighter on the ventral side, especially in specimens preserved for a longer period. 

Cuticle: Integument with secondary granules medium–small size, uniformly distributed. 

Chaetotaxy: Dorsal chaetotaxy paurochaetose, constituted by sensory, meso- and macro- chaetae, these latter well-developed in the last abdominal segments (i.e., V and VI Abd.) (Figure 2A). The dorsal chaetotaxy is similar in all populations studied, with some asymmetries and small individual differences (e.g., length of chaetae in lateral and dorsolateral areas). Thoracic tergum I with 4 + 4 chaetae, thoracic tergum II with 12 + 12 chaetae, Di with a2, De with S chaetae in p4 and Dl with 1 ms and 1 S chaeta. Thoracic tergum III as Thorax II but without a2. Abdominal terga I–IV Di with 3 chaetae and De with 4 + S chaeta in p5. Abdominal tergum V with p3 as S chaetae. Abd. VI with 2 anal spines in p1, a1 as spine-like chaetae and p0 as normal chaeta (Figure 2A,D). S chaetae formula per half tergum as 2, 2/1, 1, 1, 1 and 1. Ventral chaetotaxy shown in Figure 2C. Anal valve with 14–16 chaetae and 3 small chaetae hr (Figure 2C). Head with chaeta d0 always present; chaeta a0 variable.

Head and antennae: Antennae shorter than the cephalic diagonal. Ant. III and IV dorsally fused. Ant. I and II with 7 and 12–14 chaetae, respectively and Ant. III with 17–20 chaetae; the sensory field includes the AOIII constituted by two curved sensilla placed in a cuticular pit, two subcylindrical guard sensilla and a ventral ms (Figure 3D,E). Chaetotaxy of Ant. IV, as in Figure 3D,E, with six cylindrical sensilla (S1–4 and S7–8), a ms between S7 and S8 and a subapical organite and an apical vesicle usually trilobate (Figure 3D,E and Figure 7B,C). Ocelli 8 + 8, each group as 5 + 3 ocelli with 3 chaetae between them (Figure 2A). PAO absent. Ventral line of the head with 2 + 2 chaetae; labium with 4 + 4 chaetae in the bmf, 6 + 6 chaetae in the blf and 4 + 4 proximal chaetae and papillated chaetae L (Figure 2B); mandible with 3–4 distal and 3 basal teeth; maxilla as in *F. gretae* (Figure 6J). Labrum with 3, 5 and 4 chaetae (Figure 2B), a0 and m0 much longer than a1 and m1, respectively. m2 and a2 very lateral; 2 + 2 preclypeal chaetae.

Neotype Ratios. Body length:head diagonal:antennal length = 7.8:1.7:1.

Thorax: Chaetotaxy of the legs: subcoxae 1 of legs I, II and III with 1, 2 and 2 chaetae; subcoxae 2 of legs I, II and III, usually with 0, 3 and 3 chaetae. Coxae of legs I, II and III with 3, 8 and 8 chaetae, respectively; trochanter with 6, 6 and 6 chaetae (Figure 3C). Femora I, II and III usually with 13–14, 12 and 10–11 chaetae, respectively; variation sometimes observed. Tibiotarsi of legs I, II and III with 19, 19 and 18 chaetae, respectively, of which, 5–6 clavate, 3–4 external and 1–2 internal (Figure 3A,B and Figure 8F); distal whorl with 11 chaetae, usually one clavate chaeta on proximal whorl but sometimes other chaetae appear slightly expanded at the end; claw untoothed and empodial appendage absent.

Abdomen: Ventral tube usually 5 + 5 chaetae, one female with 4 + 5 chaetae and other with 5 + 6 chaetae; dens reduced to two small bulbs, with 5–6 chaetae on the manubrium and 3 on the dens, mucro absent; tenaculum each ramus with two teeth (Figure 8E), without chaetae on corpus. Female genital opening with 2 eugenital chaetae and ca 24–30 pregenital chaetae (Figure 3G). Male specimens with a conical papilla with 4 + 4 chaetae and about 45–58 pregenital chaetae (Figure 3F). Abdomen V with a1 as me and p2 as Mc; S chaeta only slightly longer than a1 chaeta. Abdomen VI anal spines two, short, broad at p1, plus 2 thickened spine-like chaetae at a1 and p0 as short chaetae (Figure 2A,D). Mc long, curved tip, tendency to be clavate.

Neotype Ratios. Abd V length:p2Mc:a1me:p3S = 5.5:4:1:1.2.

Neotype Ratios. Abd VI length:Mc:p1:a1 = 3:3:1:1.3.

##### Comments

Willem’s 1902 figures show well the important characters of his species. He illustrates its squat and broad habitus and dark black color, unlike *F. grisea* from South Georgia, which is more elongate and a lighter grey, and the tibiotarsus with 8 clavate tenent hairs, although he states 6 are present in the text. The furca, tenaculum, ocelli patch and dorsal abdomen VI are also well-figured. However, there are some slight errors in that he does not show the tubercular insertion of L on the labium, his ventral segment numbers are incorrect, and so he places the furca ventrally on abdomen V and the maxilla lacks one lamella. Additionally, he illustrates the mandible with 11 teeth, while we find the number to be 8.

*Friesea propria* sp. nov.: Greenslade and Fanciulli.

Type Locality: Kay Island, Victoria Land, Continental Antarctica.

Holotype: Slide FPR-KAY1, male, 26.xii.2017, A. Carapelli. Collembola Collection of the Department of Life Sciences in the University of Siena.

Paratype: Slide FPR-CCI1, female, Crater Cirque, Victoria Land, Continental Antarctica. 09. i.2019, A. Carapelli. Collembola Collection of the Department of Life Sciences in the University of Siena.

##### Material Examined for the Description

Kay Island 20 slides (6 ♀, 14 ♂); Crater Cirque 19 slides (14 ♀, 5 ♂); Tinker Glacier (10 ♀, 5 ♂) 15 slides and Harrow Peaks 6 slides (2 ♀, 4 ♂). See Table 1 for geographical details of collecting sites. Specimens will be conserved in the South Australia Museum and in the Collembola Collection of the Department of Life Sciences in the University of Siena.

Etymology: From latin, *proprius* meaning characteristic; that is, characteristic of Continental Antarctica.

Habitus Size and Color: The body length of the Holotype is 1.07 mm; the average length of specimens studied is 1.03 mm, with values ranging from 0.74 to 1.37 mm. The size is significantly smaller than *F. antarctica* (Table 3). The shape of the body is similar to this latter species, as is the color, which appears dark blue (sometimes brown dark), with some specimens lighter after longer preservation in alcohol.

Cuticle: Integument with secondary granules medium–small size, uniformly distributed. 

Chaetotaxy: *F. propria* sp. nov. shows a paurochaetose chaetotaxy both dorsally and ventrally (Figure 4A,B and Figure 7A), constituted by sensory, meso- and macro-chaetae, more developed in the last abdominal segments (i.e., V and VI Abd.); as in the previous species, some asymmetries and small individual differences were observed. Thoracic tergum I with 4 + 4 chaetae and thoracic tergum II with 12 + 12 chaetae, Di with a2, De with S chaetae in p4, Dl with 1 ms and 1 S chaeta. Thoracic tergum III as the previous one but without a2. Abdominal terga I–IV Di with 3 chaetae and De with 4 + S chaeta in p5. Abdominal tergum V with p3 as S chaeta. Abd. VI with 2 anal spines in p1, a1 as me chaeta and p0 as normal chaeta (Figure 4H and Figure 7A). S chaetae formula per half tergum as 2, 2/1, 1, 1, 1 and 1. Anal valve with 14–16 chaetae and 3 small chaetae hr (Figure 4B). On the head, chaeta d0 always present; chaeta a0 usually present (Figure 4A).

Head and Antennae: Antennae shorter than the cephalic diagonal (ratio 1.41); Ant. III and IV dorsally fused. Ant. I and II with 7 and 12–14 chaetae, respectively; Ant. III with 17–20 chaetae; the sensory field includes the AOIII constituted by two curved sensilla placed in a cuticular pit, two subcylindrical guard sensilla and a ventral ms. Chaetotaxy of Ant. IV with six cylindrical sensillae (S1–4 and S7–8), a ms between S7 and S8, a subapical organite and an apical vesicle, usually trilobate. Ocelli 8 + 8, each group as 5 + 3 ocelli with 3 chaetae between them (Figure 4A and Figure 7E). PAO absent. Ventral line of the head with 2 + 2 chaetae; labium with 4 + 4 chaetae in the bmf, 6 + 6 chaetae in the blf and 4 + 4 proximal chaetae and papillated chaetae L (Figure 8C); mandible with 3–4 distal and 3 basal teeth; maxilla as in *F. gretae* (Figure 6J). Labrum with 3, 5 and 4 chaetae and a0 and m0 much longer than a1 and m1, respectively; m2 and a2 very lateral and 2 + 2 preclypeal chaetae.

Thorax: Chaetotaxy of the legs: subcoxae 1 of legs I, II and III with 1, 2 and 2 chaetae; subcoxae 2 of legs I, II and III, usually with 0, 2 and 2 chaetae. Coxae of legs I, II and III with 3, 8 and 8 chaetae; trochantera I, II and III with 6 chaetae each (Figure 4E). Femora I, II and III, usually with 13–14, 12 and 10–11 chaetae, respectively; variations sometimes observed. Tibiotarsi of legs I, II and III with 19, 19 and 18 chaetae, of which 5–6 clavate, usually 3–4 external and 1–2 internal (Figure 4F,G); distal whorl with 11 chaetae; also, in this species, as in *F. antarctica*, variability in the distal end shape of clavate chaetae was observed; claw untoothed and empodial appendage absent (Figure 4F,G).

Abdomen: Ventral tube usually 4 + 4 chaetae, dens reduced to two small bulbs, with 5–6 chaetae on the manubrium and 3 on the dens and mucron absent; tenaculum each ramus with two teeth, without chaetae on corpus. Female genital opening with 2 eugenital chaetae and ca 11–22 pregenital chaetae (Figure 4D). Male specimens with a conical papilla with 4 + 4 chaetae and about 21–30 pregenital chaetae (Figure 4C and Figure 8A). 

Abdomen V with a1 as me and p1 as Mc; p3 as sensory chaeta; p1 and p3S usually of the same length but longer than a1 (Figure 4H). Ratio Abd.V:p1Mc:a1me:p3S = 3.5:1.5:1:1.5.

Abdomen VI anal spines two, short and broad at p1, me chaeta at a1; p0 short chaeta (Figure 4H).

*Friesea gretae* sp. nov.: Greenslade and Fanciulli.

Type Locality: Cape Hallett.

Holotype: Slide C, female, K140I, 18c, 16.xii.2002, B. Sinclair. South Australia Museum.

Paratype: Slide B, female, Cape Hallett, West side Hallett Inlet, Dry Hanging Valley 1-3.xii.1959, Brian Reid South Australia Museum.

##### Material Examined for the Description

Cape Hallett, North Victoria Land, Continental Antarctica, 22 slides (12 ♀, 10 ♂). See Table 1 for geographical details of collecting sites. Specimens will be conserved in the South Australia Museum and in the Collembola Collection of the Department of Life Sciences in the University of Siena.

Etymology: Named after Greta Tintin Eleonora Ernman Thunberg, young advocate for action on climate change.

Habitus Size and Color: The body length of the holotype is 0.95 mm; the average length of specimens studied is 1.28 mm, with values ranging from 0.95 to 1.60 mm. The size of *F. gretae* sp. nov. is significantly smaller than *F. antarctica* and larger than of *F. propria* (Table 3); the shape of the body (especially in the specimens preserved in alcohol before the preparation of the slides) appears cylindrical, slenderer than those in *F. antarctica* and *F. propria*.

Cuticle: Integument with uniformly distributed medium–small size granules, without any plaque or tubercles along the body. 

Chaetotaxy: *F. gretae* n. sp. also shows a paurochaetose chaetotaxy both dorsally and ventrally (Figure 5A,B,D), constituted by sensory, meso- and macro-chaetae, more developed in the last abdominal segments (i.e., V and VI Abd.); as in the previous species, some asymmetries and small individual differences were observed. Thoracic tergum I with 4 + 4 chaetae and thoracic tergum II with 12 + 12 chaetae, Di with a2, De with S chaetae in p4, Dl with 1 ms and 1 S chaeta. Thoracic tergum III as the previous one but without a2. Abdominal terga I–IV Di with 3 chaetae and De with 4 + S chaeta in p5. Abdominal tergum V with p3 as S chaetae. Abd. VI with 2 anal spines in p1, a1 as spiny chaeta and p0 as me chaeta (Figure 5A,E). S chaetae formula per half tergum as 2, 2/1, 1, 1, 1 and 1. Anal valve with 14–16 chaetae and 3 small chaetae hr (Figure 5D). Head with chaeta d0 always present; chaeta a0 usually present. 

Head and Antennae: Antennae shorter than the cephalic diagonal (ratio 1.39); Ant. III and IV dorsally fused. Ant. I and II with 7 and 12–14 chaetae, respectively (Figure 6F); Ant. III with 17–20 chaetae; the sensory field includes the AOIII constituted by two curved sensilla placed in a cuticular pit, two subcylindrical guard sensilla and a ventral ms (Figure 6E and Figure 7D). Chaetotaxy of Ant. IV as in Figure 6D,E with six cylindrical sensilla (S1–4 and S7–8), a ms between S7 and S8, a subapical organite and an apical vesicle, usually trilobate. Ocelli 8 + 8, each group as 5 + 3 ocelli with 3 chaetae between them. PAO absent. Ventral line of the head with 2 + 2 chaetae; labium with 4 + 4 chaetae in the bmf, 6 + 6 chaetae in the blf and 4 + 4 proximal chaetae and papillated chaetae L (Figure 5C); mandible with 3–4 distal and 3 basal teeth (Figure 6I); maxilla as in Figure 6J. Labrum with 3, 5 and 4 chaetae and a0 and m0 much longer than a1 and m1, respectively; m2 and a2 very lateral, 2 + 2 preclypeal chaetae (Figure 5C).

Thorax: Chaetotaxy of the legs: subcoxae 1 of legs I, II and III with 1, 2 and 2 chaetae; subcoxae 2 of legs I, II and III, usually with 0, 2 and 2 chaetae. Coxae of legs I, II and III with 3, 8 and 8 chaetae; trochantera I, II and III with 6, 6 and 6 chaetae each (Figure 6C). Femora I, II and III, usually with 13–14, 12 and 10–11 chaetae, respectively; variations sometimes observed. Tibiotarsi of legs I, II and III with 19, 19 and 18 chaetae, of which 5–6 clavate, usually 3–4 external and 1–2 internal (Figure 6A,B); distal whorl with 11 chaetae; usually one clavate chaeta on proximal whorl; claw untoothed and empodial appendage absent (Figure 6A,B).

Abdomen: Ventral tube usually 4 + 4 chaetae and dens reduced to two small bulbs, with 5–6 chaetae on the manubrium and 3 on the dens, mucron absent; tenaculum each ramus with two teeth, without chaetae on corpus (Figure 6K and Figure 8D). Female genital opening with 2 eugenital chaetae and ca. 11–14 pregenital chaetae (Figure 6H and Figure 8B). Male specimens with a conical papilla with 4 + 4 chaetae and ca. 15–22 pregenital chaetae (Figure 6G). Abdomen V with a1 as me and p1 as spiny Mc; p3 as sensory chaeta; p1 and p3S usually of the same length but longer than a1 (Figure 5E). Ratio Abd.V:p1Mc:a1me:p3S = 3.6:1.4:1:1.4. Abdomen VI anal spines two, short and broad at p1, spiny chaeta at a1 and p0 short chaetae (Figure 5E).

### 3.2. Phylogenetic Analyses

**Figure 9:**
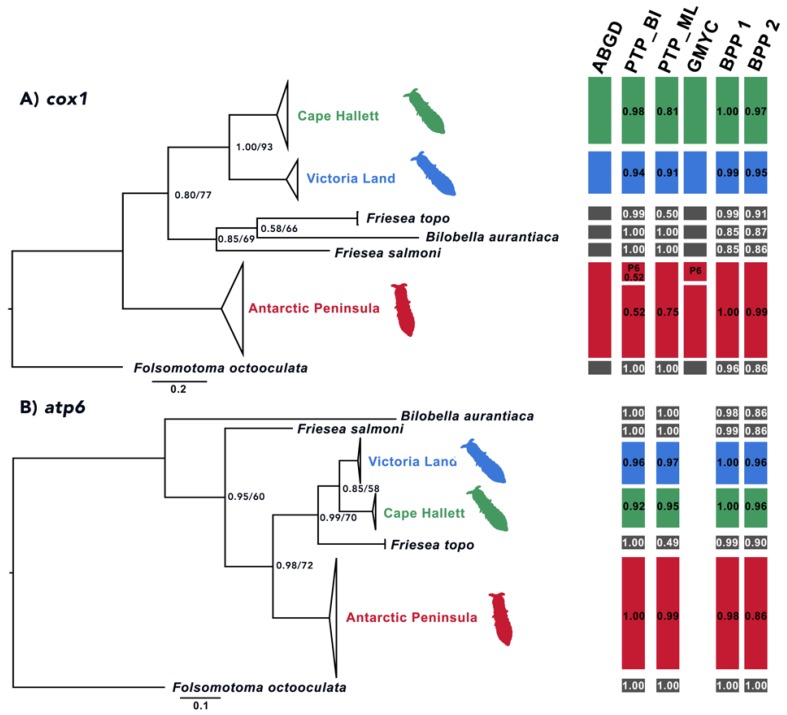
The phylogenetic tree of the *Friesea* lineages under study obtained through Bayesian (BI) and maximum likelihood (ML) inference methods, applying the: (**A**) cytochrome *c* oxidase subunit one (*cox1*) dataset and (**B**) the ATP synthase subunit six (*atp6*) datasets. Bootstrap values/posterior probabilities indicated only when both do not correspond to maximum values. In red, the cluster of the 14 populations sampled along the Antarctic Peninsula; in blue, the phylogroup of the seven populations collected in Continental Antarctica and in green, the branch including six haplotypes from Cape Hallett. Outgroup species listed in black. On the right, a summary of the species delimitation methods performed on the single-locus dataset. Black boxes indicate the outgroup species, whereas red, blue and green identify samples from the alternative geographical regions (described as above). Applied methods of species delimitation listed above bars as follows: ABGD, automatic barcode gap discovery; PTP, Poisson tree process applying BI or ML topology, respectively, and including the outgroup species; GMYC, generalized mixed yule coalescent model and BPP, Bayesian phylogenetics and phylogeography, run with priors θ:G (1:10), τ:G (1:10) (BPP1) and θ:G (2:100), τ:G (2:500) (BPP2). For species distributions, see Discussion.

**Figure 10:**
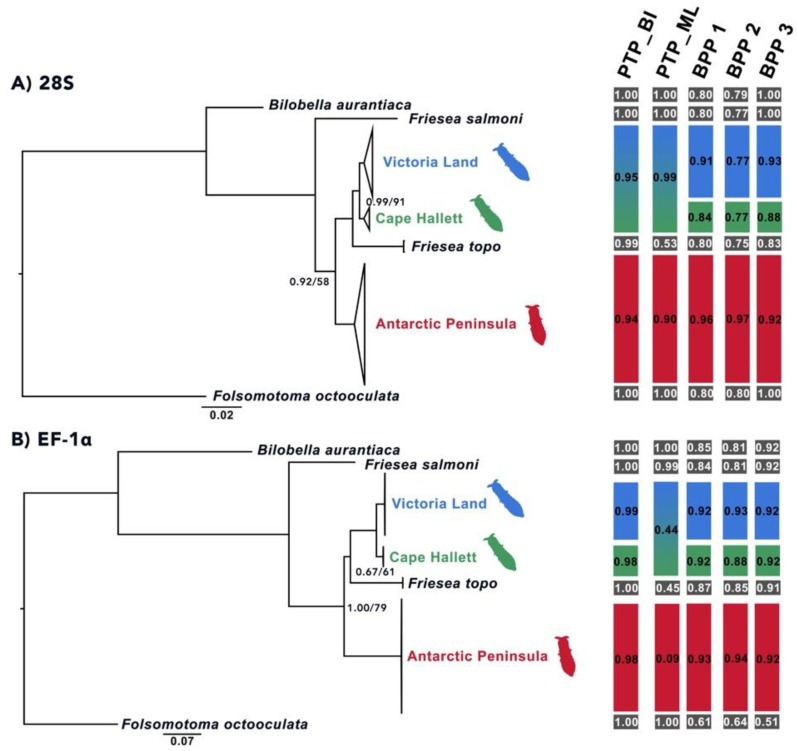
The phylogenetic tree of the *Friesea* lineages under study obtained through Bayesian (BI) and maximum likelihood (ML) inference methods, applying the: (**A**) large nuclear rRNA gene (*28S*) dataset and (**B**) the elongation factor 1α (*EF-1α*) datasets. Bootstrap values/posterior probabilities indicated only when both do not correspond to maximum values. In red, the cluster of the 14 populations sampled in Antarctic Peninsula sites; in blue, the phylogroup of the seven populations collected in the Continental Antarctica and in green, the branch including the two *28S* and the single *EF-1α* variants detected from Cape Hallett. Outgroup species listed in black. On the right, a summary of the species delimitation methods performed on the single-locus dataset. Black boxes indicate the outgroup species, whereas red, blue and green identify samples from the alternative geographical regions (described as above). Applied methods of species delimitation listed above bars as follows: ABGD, automatic barcode gap discovery; PTP, Poisson tree process applying BI or ML topology, respectively, and including the outgroup species and BPP, Bayesian phylogenetics and phylogeography, run with priors: θ:G (1:10), τ:G (1:10) (BPP1), θ:G (2:100), τ:G (2:500) (BPP2) and θ:G (2:1000), τ:G (2:100) (BPP3). For species distributions, see Discussion.

**Figure 11:**
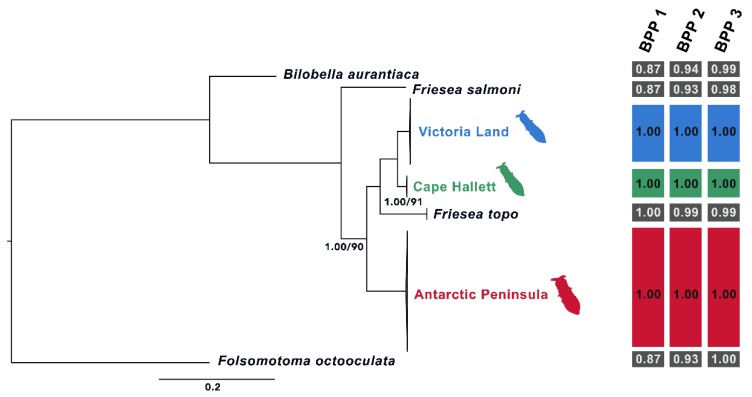
The phylogenetic tree of the *Friesea* lineages under study obtained through Bayesian (BI) and maximum Likelihood (ML) inference methods, applying the multi-locus dataset (combined alignment of *cox1*, *atp6*, *28S* and *EF-1α*. Bootstrap values/posterior probabilities indicated only when both do not correspond to maximum values. In red, the cluster of the 14 populations sampled in Antarctic Peninsula sites; in blue, the phylogroup of the seven populations collected in the Continental Antarctica and in green, the branch including three specimens from Cape Hallett. Outgroup species listed in black. On the right, a summary of the species delimitation analyses performed using the multi-locus dataset. Black boxes indicate the outgroup species, whereas red, blue and green identify samples from the alternative geographical regions (described as above). Applied method of species delimitation Bayesian phylogenetics and phylogeography, run with priors: θ:G (1:10), τ:G (1:10) (BPP1), θ:G (2:100), τ:G (2:500) (BPP2) and θ:G (2:1000), τ:G (2:100) (BPP3). For species distribution, see Discussion paragraph.

The single locus phylogenetic trees obtained through both BI and ML showed a clear distinction between three main *F. antarctica* lineages (AP, VL and Cape Hallett), with moderate to high statistical support (PP = 0.92–1.00; BS = 58–79; Figure 9 and Figure 10). In all single locus BI and ML topologies, except that based on the *cox1* dataset, *F. topo* (specimens collected on Alexander I.) is basal to VL and Cape Hallett samples and not to *F. antarctica* from the AP, as simple geography would suggest. The same phylogenetic relationships are obtained when the four-locus matrix is used for comparison (Figure 11). In this latter instance, all the nodes were strongly supported, further suggesting that the three *F. antarctica* clusters may be well-defined different evolutionary entities. In addition, when the amplification of the 28S rRNA-encoding gene was performed using the primer pair D1a/D7b, one single amplification product (of ~2100 bp) was obtained from almost all samples, with the exception of those of *F. antarctica* from VL (seven specimens from as many localities but not from Cape Hallett; Table 1), where two bands of different size were coamplified and sequenced. Of these, the largest band was clearly homologous to the single amplified product that was obtained from the AP and Cape Hallett samples and corresponds to the complete and expected gene-fragment size; the smaller probably corresponds to a *28S* rRNA pseudogene representing a copy missing more than 400 bp. Attempts to amplify the smaller band in specimens collected elsewhere from Victoria Land and in those from Cape Hallett were unsuccessful, suggesting that the pseudogene copy may have evolved only in the ancestor of this *F. antarctica* group, for which it could be considered a synapomorphic character.

### 3.3. Species Delimitation Analyses

Regardless of the species delimitation method applied, all the discovery approaches tested suggested that *F. antarctica* should be considered a species complex. The lineages corresponding to AP, VL and Cape Hallett were identified as different taxa by most of the discovery methods applied to the single-locus datasets. Overall, the statistical support obtained for each of these hypotheses was moderate to high (Figure 9 and Figure 10). Few exceptions to this pattern were observed. First, when the PTP method was applied to the BI topology of the *cox1* dataset and the ultrametric tree was used in the GMYC analysis, the AP lineage was split into two clusters: one including only the haplotype P6 and the other including all the remaining *cox1* sequences. Second, the PTP method applied to the ML topology of the *EF-1α* dataset suggested that the VL and Cape Hallett lineages should be considered as a single species. However, this analysis provided low statistical support to the single species hypothesis for the continental Antarctic (i.e., VL + Cape Hallett, PP = 0.44) and for the AP (PP = 0.09; Figure 11). Third, the application of the PTP method to the *28S* dataset proposed VL and Cape Hallett lineages as a single species with high posterior probability values (Figure 10A). This outcome was observed applying both the BI and ML topology.

The validation method of species delimitation was employed giving as prior information the presence of three different species corresponding to the AP, VL and Cape Hallett lineages, since most of the discovery analyses supported this hypothesis. During the validation analyses, all the possible combinations of species were further tested, and the posterior probability of a given delimitation was calculated.

In all instances (single- and multi-locus datasets, as well as the three combinations of θ and τ parameters), the presence of three distinct species was robustly supported (PP between 0.70 and 1.00; Figure 9, Figure 10 and Figure 11). When the existence of one species (AP + VL + Cape Hallett) or two (AP vs. VL + Cape Hallett) was suggested, the posterior probability values were very low (all PP < 0.01; data not shown). The only ambiguous output was obtained with a combination of θ:G (2:1000), τ:G (2:200) parameters applied to the *cox1* and *atp6* single-locus datasets; this output suggests the existence of a single species including all three *F. antarctica* lineages plus the outgroups, a suggestion that is obviously not tenable (data not shown).

## 4. Discussion

The application of an integrated taxonomy approach, here tested for springtail species, appears to be useful in delimiting species boundaries in cases in which morphological diagnostic characters are ambiguous [56,57]. A similar method has recently been applied to Antarctic terrestrial nematodes and was successful in detecting nematode lineages in the Maritime Antarctic [58]. The present study further confirms the efficacy of an integrated taxonomic approach to better define and characterize different ecosystem biodiversity.

Morphological analyses have thus far struggled to find diagnostic characters among different local forms of *F. antarctica*. On the other hand, the morphological reassessment performed herein in light of the outcome of the molecular analysis led to the identification of some characters that, though marginal, are consistent with the molecular results. Specifically, one character (setae in subcoxa 2) is fixed as 0-3-3 in populations from the Antarctic Peninsula and as 0-2-2 in populations from Victoria Land (inclusive of Cape Hallett). Three other characters (body length, setae on the male and female genital opening) are quantitative in nature but display a statistically significant difference among groups (Table 3 and Table 4).

The analysis of mitochondrial, as well as nuclear markers, provided consistent results, as well as supporting the efficacy of the barcode *cox1* gene in the identification of species boundaries. The only exception to this outcome was represented by the *28S*, which, at variance, supported the presence of a single species from the Continental Antarctic (VL + Cape Hallett; Figure 10A). We can hypothesize that the latter two taxa (i.e., VL and Cape Hallett) started to diverge more recently, and therefore, that the large ribosomal subunit may have not yet accumulated sufficient mutations to discriminate between the two lineages.

Nevertheless, during the amplification of the *28S* gene (portion between D1 and D7), the presence of two copies of the large ribosomal subunit was observed which differ by about 470 bp in length. This feature was present only in the specimens of *F. antarctica* sampled in Victoria Land, with the exception of those from Cape Hallett. These latter, together with the specimens from AP, possessed only the longer copy of the *28S*. All phylogenetic reconstructions support *F. topo* as basal to an assemblage of *F. antarctica* populations from Victoria Land (inclusive of Cape Hallett), with the exclusion of *F. antarctica* populations from the Antarctic Peninsula. This would make *F. antarctica* as currently defined polyphyletic, while it is consistent with the proposed subdivision of *F. antarctica* into different lineages.

The sharp genetic distinction between the three *F. antarctica* lineages is not striking *per se*, as recent studies already put forward the hypothesis that *F. antarctica* may represent a cluster of multiple cryptic species [4,35]. In fact, striking was the previous recognition of the presence over Antarctica of a single “pan-Antarctic” springtail species, especially considering that most of the sampling sites correspond to physically remote geographic regions (Antarctic Peninsula and Victoria Land) and also taking into account the vicariant origin of Antarctic springtails in general [59,60] and their limited dispersal capabilities. This latter characteristic is likely to underlie the differentiation of a third evolutionary lineage in Cape Hallett, the northern-most sampling locality along the Victoria Land coastline, which is separated from all the other sites by the major barrier of the Tucker Glacier (Figure 1 and Table 1). This geographic barrier seems to be insurmountable for *F. antarctica,* as has already been shown to be for other springtail species endemic to Victoria Land (*K. klovstadi* and *C. cisantarcticus*) that show high levels of genetic divergence between populations from either side of the Tucker Glacier (e.g., [4]). This level of fragmentation raises significant conservation issues. This is particularly the case for *F. gretae*, which is currently known only from its type locality at Cape Hallett and is now one of three terrestrial micro-arthropods whose type locality is this location (Antarctic Treaty Secretariat, 2015, available at http://documents.ats.aq/recatt%5Catt567_e.pdf). These occur within an Antarctic specially protected area (ASPA No. 106), an area declared both to protect a significant Adélie penguin population and in recognition of its exceptional vegetation development and terrestrial biodiversity by regional standards. This ASPA, however, faces the risk of long-term disturbance to its terrestrial habitats due to human research activities and, in particular, through the very limited options available for erecting and operating field camps. As highlighted by [30], the agreed management plan of this ASPA requires updating, as the primary recommended camping area overlaps with the known distribution of this new species of endemic springtail. Protection of “the type locality or only known habitat of any species” is listed as one of the primary criteria supporting the declaration of areas as ASPAs (Annex V, Article 3(2) of the Environmental Protocol to the Antarctic Treaty) (https://www.environments.aq/information-summaries/specially-protected-and-managed-areas-in-antarctica-updated/).

The highly conserved morphology observed between the three putative species suggests the occurrence of morphological stasis (or phylogenetic niche conservatism), a phenomenon that arises when a species is under particularly strong selective pressures (adversity selection [61]). In a context such as the extreme Antarctic terrestrial environment, the selection of particular characters is greatly influenced by abiotic factors that, over Antarctica, are the major forces shaping terrestrial biota [19,62,63]. Among them, freezing temperatures, drought and exposure to high levels of UV radiation would likely support the selection of darker pigmentation and smaller body size, as well as earlier sexual maturity due to the very short active season [3]. In this context, the significant difference in body size and the reduced number of setae in the genital openings observed between *F. antarctica* lineages may be considered of substantial biological importance.

## 5. Conclusions

The Antarctic Continent and Peninsula environments are predictably severe, a defining characteristic for adversity selection [61,62], which would be here operating on organisms. In this type of selection, morphological stasis is the norm, because the common drivers of morphological adaptation, such as biotic and climatic variability, are low or absent. Competition and predator pressures are low because of extremely low species numbers and the types of habitats present are few. In habitats where adversity selection operates, there is selection against dispersal and for any change in the organism, because it is already highly adapted to severe conditions. Consequently, it is not then surprising that morphological changes in the *Friesea* species group in Antarctica has been extremely slow over long time periods. The same phenomenon is found in other Antarctic springtail genera, such as the genus *Tullbergia* [64].

The identification of one single springtail species whose distribution spans areas as distant as Victoria Land and the Antarctic Peninsula, contrasting with all other Antarctic species that are restricted to a single region, together with its fairly high levels of “intra-specific” genetic divergence, has puzzled researchers for over two decades. Nevertheless, hindered by a marked inter-individual population-level variability in the context of an otherwise morphologically uniform taxon, classical morphological taxonomy had thus far failed to find evidence for multiple species.

In this study, a massive sampling effort and multi-locus molecular data and phylogenetic analyses, as well as species delimitation methods, were applied to the study of *F. antarctica* diversity on a continental scale. These led to the identification of three well-defined and distinct lineages. Based on this initial hypothesis, the morphology of specimens from the entire continent was revised, and a number of subtle morphological characters were identified that provide further support for the three lineages being species-level entities: one corresponds to *F. antarctica* and two are here formally described as *F. gretae* nov. sp. and *F. propria* nov. sp. From a methodological standpoint, we underline the strength of the combined application of molecular, biogeographic and morphological data, an approach that has gained momentum in the last 10–15 years as “integrated taxonomy”. From the point-of-view of Antarctic Collembolan diversity, this study resolves the enigma of the uniqueness of a single “pan-Antarctic” species and provides a well-supported scenario leading to a formal taxonomic assessment of the three species.

## Figures and Tables

**Table 1:** Overview of sampling localities, with respective site codes and location coordinates; the haplotypes/alleles detected in each population are shown. Asterisks highlight the populations also included in the morphological analyses; in red are new sequences obtained in the present study.

Collection Site	Label	Coordinates	Haplotypes/Alleles			
			*cox1*	*atp6*	*28S*	*EF-1α*
**Antarctic Peninsula—AP**						
Potter Cove, King George Is.	PCK *	62°14’ S, 58°42’ W	P3	P1	P1	P1
Rip Point, Nelson Is.	RPN	62°15’ S, 58°59’ W	P3	P1	P3	P1
Harmony Point, Nelson Is.	HPN *	62°19’ S, 59°10’ W	P1, P2, P3	P1, P2, P3	P1	P1
Coppermine Peninsula, Robert Is.	CPR *	62°23’ S, 59°42’ W	P3	P1, P4, P5	P6	P1
Hannah Point, Livingston Is.	HAL	62°39’ S, 60°36’ W	P3, P5, P6	P1, P8, P9, P10	P1	P1
Devils Point, Livingston Is.	DBL *	62°40’ S, 61°11’ W	P7	P11	P2	P1
Hurd Peninsula, Livingston Is.	HUL *	62°41’ S, 60°23’ W	P3, P4	P1, P6, P7	P1	P1
Danco Is.	DAI	64°44’ S, 62°37’ W	P8	P14	P1	P1
Detaille Is.	DEI	66°52’ S, 66°47’ W	P8	P13	P1	P1
Mackay Point, Adelaide Is.	MPA	67°32’ S, 68°04’ W	P3	P12	P5	P1
Killingbeck Is.	KII	67°34’ S, 68°04’ W	P3	P12	P4	P1
North Point Rothera, Adelaide Is.	NRA *	67°34’ S, 68°06’ W	P3	P12	P5	P1
Reptile Ridge, Adelaide Is.	RRA *	67°33’ S, 68°11’ W	P3	P12	P1	P1
Lagoon Is.	LIS *	67°35’ S, 68°14’ W	P1, P3	P12	P1	P1
**Victoria Land—VL**						
Cape Hallett	CHA *	72°25’ S, 169°58’ E	V1, V2, V3, V4, V5, V6	V1, V2, V3, V4, V5, V6	V7, V8	V2
Crater Cirque	CCI *	72°38’ S, 169°37’ E	V7, V8	V7, V8, V9	V5	V1
Cape King	CKI	73°35’ S, 166° 37’ E	V7	V9	V4	V1
Hayes Head	HHE	74°01’ S, 165°18’ E	V7	V9	V2	V1
Tinker Glacier 2	TG2	74°02’ S, 165°04’ E	V7	V9	V1	V1
Kay Is.	KAI *	74°04’ S, 165°19’ E	V7, V9, V10	V9	V6	V1
Tinker Glacier 1	TG1 *	74°02’ S, 164°49’ E	V7	V9, V10, V11	V1	V1
Harrow Peaks	HPE *	74°06’ S, 164°48’ E	V7	V9	V3	V1

**Table 2:** List of the models of evolution that best fit our datasets, divided according to the dataset (single and multi-locus), to the partition applied and to the respective tree search optimization criteria.

	Bayesian Inference				Maximum Likelihood			
**Single-Locus**	**1st**	**2nd**	**3rd**	**Non-Cod.**	**1st**	**2nd**	**3rd**	**Non-Cod.**
*cox1*	GTR+I	GTR	HKY+Г	-	TPM3u+F	TPM2+F+G4	TIM2e+I	-
*atp6*	GTR+Г	GTR+Г	GTR+Г		TN+F+I	F81+F+G4	TN+F+G4	-
*EF-1α*	F81+I	F81+I	GTR+Г	HKY+I	TPM2+F+G4	F81+F+I	F81+F+I	HKY+F+I
*28S*	-	-	-	GTR+Г	-	-	-	TVM+F+G4
**Multi-locus**	**1st**	**2nd**	**3rd**	**Non-Cod.**	**1st**	**2nd**	**3rd**	**Non-Cod.**
*cox1*	GTR+I	HKY	HKY+Г	-	TIM2e+I	F81+F	HKY+F+G4	-
*atp6*	GTR+I	F81+Г	HKY+Г	-	TN+F+I	F81+F+G4	HKY+F+G4	-
*EF-1α*	GTR+I	GTR+I	GTR+Г	HKY+Г	F81+F	F81+F	TPM2u+F	HKY+F
*28S*	-	-	-	GTR+Г	-	-	-	TVM+F+G4

**Table 3:** The body length (expressed in mm), the ratio cephalic diagonal/antennae, the ratio length/width of each thoracic and abdominal segment and the number of setae in the male and female genital openings calculated for each putative species, and compared as: (a) Antarctic Peninsula vs. Victoria Land (excluding Cape Hallett), (b) Antarctic Peninsula vs. Cape Hallett and (c) Victoria Land vs. Cape Hallett only. The number of specimens evaluated and parameter means, ranges and standard deviations are shown. Significant (*p* < 0.05 after Bonferroni correction) differences are highlighted.

***F. antarctica* Antarctic Peninsula (AP)**	**n**	**Mean**	**Range**	**Standard Deviation**	**Different from**
Body length	45	1.55	1.16–2.00	0.22	VL, CHA
Ratio cephalic diagonal/antenna	8	1.40	1.17–1.64	0.14	
Th.I	8	0.27	0.24–0.33	0.03	
Th.II	8	0.46	0.39–0.54	0.05	CHA
Th.III	8	0.50	0.43–0.56	0.04	
Abd.I	8	0.38	0.30–0.47	0.05	
Abd.II	8	0.36	0.26–0.44	0.05	
Abd.III	8	0.34	0.28–0.39	0.04	
Abd.IV	8	0.38	0.35–0.44	0.04	
Abd.V	8	0.41	0.37–0.45	0.03	
Abd.VI	8	0.61	0.48–0.67	0.06	
Setae male genital opening	20	48.40	36–59	7.58	VL, CHA
Setae female genital opening	12	26.70	15–30	4.18	VL, CHA
***F. propria* Victoria Land (VL) excluding Cape Hallett**					
Body length	64	1.03	0.74–1.37	0.17	CHA, AP
Ratio cephalic diagonal/antenna	12	1.41	1.24–1.70	0.14	
Th.I	12	0.32	0.19–0.40	0.06	
Th.II	12	0.49	0.38–0.66	0.07	
Th.III	12	0.53	0.40–0.67	0.08	
Abd.I	12	0.39	0.23–0.48	0.07	
Abd.II	12	0.35	0.26–0.45	0.05	
Abd.III	12	0.34	0.25–0.45	0.06	
Abd.IV	12	0.40	0.29–0.54	0.07	
Abd.V	12	0.38	0.37–0.45	0.06	CHA
Abd.VI	12	0.64	0.57–0.81	0.07	
Setae male genital opening	14	25.36	21–32	2.84	CHA, AP
Setae female genital opening	17	15.41	10–22	3.35	AP
***F. gretae* Cape Hallett**					
Body length	17	1.28	0.95–1.60	0.18	VL, AP
Ratio cephalic diagonal/antenna	6	1.39	1.31–1.51	0.07	
Th.I	6	0.43	0.36–0.49	0.06	
Th.II	6	0.62	0.59–0.69	0.04	AP
Th.III	6	0.67	0.62–0.80	0.06	
Abd.I	6	0.53	0.36–0.62	0.08	
Abd.II	6	0.44	0.41–0.49	0.03	
Abd.III	6	0.40	0.29–0.48	0.06	
Abd.IV	6	0.49	0.45–0.53	0.02	
Abd.V	6	0.51	0.45–0.55	0.04	VL
Abd.VI	6	0.72	0.60–0.80	0.06	
Setae male genital opening	6	20.67	20–22	1.03	VL, AP
Setae female genital opening	8	12.37	11–15	1.60	AP

**Table 4:** Morphological characters supporting the subdivision of *Friesea antarctica* into three species. Numbers, where appropriate, indicate mean (minimum–maximum) or range. +/− signs indicate presence/absence of specific chaetae. Spiny/Meso refer to the shape of the a1 chaeta on Abd. VI. Chaetae ratios are reported as Abd.V length: p2 (p1): a1: p3S. p2 is used for *Friesea antarctica* and p1 for *Friesea propria* sp. nov. and *Friesea gretae* sp. nov.

	*Friesea antarctica*	*Friesea propria* sp. nov.	*Friesea gretae* sp. nov.
Body length	1.53 (1.15–2.00)	1.04 (0.79–1.37)	1.28 (0.95–1.60)
Chaetae male genital opening	36–59	21–32	20–22
Chaetae female genital opening	15–30	10–22	11–15
Chaetae subcoxa 2	0-3-3	0-2-2	0-2-2
Chaetae on ventral tube	5 + 5	4 + 4	4 + 4
Abd. V p1 chaeta	−	+	+
Abd. V p2 chaeta	+	−	−
Abd. VI a1 chaeta	spiny	meso	spiny
Abd V chaetae ratios	5.5:4:1:1.2	3.5:1.5:1:1.5	3.6:1.4:1:1.4.

## Abbreviations

a	anterior/apical chaeta
Abd	abdomen;
ant	antenna
blf	basolateral field
bmf	basomedian field
d	dorsal chaeta
De	dorso-external area
Di	dorso-internal area
DL	dorso-lateral area
L	lateral area
Mc	macrochaeta
me	mesochaeta
ms	microsensillum
p	posterior chaeta
PAO	postantennal organ
S	sensorial chaeta
th	thorax
